# Physiological and Transcriptional Responses of Sesame (*Sesamum indicum* L.) to Waterlogging Stress

**DOI:** 10.3390/ijms26062603

**Published:** 2025-03-13

**Authors:** Yadong Fan, Chengqi Cui, Yanyang Liu, Ke Wu, Zhenwei Du, Xiaolin Jiang, Fengli Zhao, Ruping Zhang, Jingjing Wang, Hongxian Mei, Haiyang Zhang

**Affiliations:** 1 Henan Sesame Research Center, Henan Academy of Agricultural Sciences, Zhengzhou 450002, China; fyd308@163.com (Y.F.); sesame_ccq@126.com (C.C.); liuyanyang001@163.com (Y.L.); wking2010@yeah.net (K.W.); duzhw123@163.com (Z.D.); jiangxl2006@126.com (X.J.); fenglizhao1990@126.com (F.Z.); zrpzrpzrp@126.com (R.Z.); jingle_93@163.com (J.W.); 2 The Shennong Laboratory, Zhengzhou 450002, China

**Keywords:** sesame, waterlogging, transcriptome

## Abstract

Waterlogging stress significantly impacts the growth and productivity of crops. As a traditional oil crop, sesame (*Sesamum indicum* L.) suffers substantial damage due to waterlogging stress. However, the mechanism underlying waterlogging stress in sesame is still unclear. In this study, we investigated the physiological indicators of two sesame genotypes under waterlogging stress. The results revealed that the activity of antioxidant enzymes in sesame was affected, with the contents of malondialdehyde (MDA) and hydrogen peroxide (H_2_O_2_) significantly increased. Additionally, transcriptional analysis identified a total of 15,143 differentially expressed genes (DEGs). Among them, 759 DEGs exhibited consistent differential expression across all time points, representing the core waterlogging-responsive genes. Gene Ontology (GO) enrichment analysis indicated that the DEGs were primarily associated with hypoxia, stimulus response, and oxidoreductase enzyme activities. Kyoto Encyclopedia of Genes and Genomes (KEGG) analysis revealed that these DEGs were mainly enriched in the metabolic and biosynthesis of secondary metabolites, glycolysis/gluconeogenesis, phenylpropanoid biosynthesis, MAPK signaling pathway-plant, carbon fixation by Calvin cycle, plant hormone signal transduction, and plant-pathogen interaction pathways. Furthermore, transcription factors (TFs) such as AP2/ERF, bHLH, bZIP, and WRKY may play key roles in the transcriptional changes induced by waterlogging stress. Combined with weighted gene co-expression network analysis (WGCNA) analysis and K-means clustering, a total of 5 hub genes and 56 genes were identified, including F-box protein (*Sin09950* and *Sin12912*), bZIP (*Sin04465*, *Sin00091*), WRKY (*Sin01376*, *Sin06113*), and so on. In brief, this study explored the regulatory network involved in waterlogging stress in sesame at the transcriptome level, providing valuable insights into unraveling the molecular mechanisms of waterlogging stress and facilitating the breeding of improved waterlogging-tolerant sesame varieties.

## 1. Introduction

With the climate changes, floods are intensifying and becoming more frequent, putting further pressure on agricultural security [1]. As one of the most frequent and catastrophic climate-related hazards, floods have a severe impact on agriculture as well as other sectors. Floods inflict the second largest impact, following extreme temperatures and droughts, with a responsible loss reaching about 16–20% [2,3]. Flooding refers to the excess water replacing gas spaces surrounding the plants. Depending on soil water level conditions, it can also be called waterlogging and submergence [4,5]. Most crops are sensitive to waterlogging stress and intolerant of complete submergence, even rice [6]. Waterlogging results in deficient soil oxygen levels, hindering plant root respiration, and triggering a series of physiological changes such as oxidative damage, accumulation of toxic metabolites, obstruction of nutrient transport, and decline in photosynthetic capacity, seriously affecting plant growth and development [7]. To cope with waterlogging stress and mitigate damage, plants have evolved two adaptive mechanisms: low-oxygen escape syndrome (LOES) and low-oxygen quiescence syndrome (LOQS). Under LOES, plants escape waterlogged environments by elongating their above-ground parts, forming aerenchyma, and developing adventitious roots. Unlike the energy-consuming LOES, LOQS maintains cellular homeostasis by altering metabolic pathways and inhibits plant growth and development [8,9,10,11].

In the biological process of plants responding to waterlogging stress, involved complex signal transduction and gene expression regulation, which are extremely important for plants [8]. Waterlogging stress induces oxidative damage, producing a large amount of reactive oxygen species (ROS), including hydrogen peroxide (H_2_O_2_), superoxide anion (O_2_^−^), and other metabolites [12]. Plants can counteract the adverse effects of hypoxia stress through their inherent antioxidant systems by enhancing the activities of antioxidant enzymes, including catalase (CAT), peroxidase (POD), ascorbate peroxidase (APX), superoxide dismutase (SOD), and glutathione peroxidase (GSH-Px), as well as non-enzymatic antioxidants like ascorbic acid (AsA), glutathione (GSH), and melatonin [5,13,14]. Moreover, physiological indicators revealed increased levels of soluble sugars, proteins, proline, and malondialdehyde (MDA) content under waterlogging stress. Soluble sugars not only function as a vital source of energy and carbon but also play a crucial role in maintaining osmotic balance within cells, often reflecting the resistance of plants. Proline is an important reactive oxygen species scavenger, and its accumulation in plants is considered one of the adaptive responses to adversity. As a product of lipid peroxidation, malondialdehyde (MDA) is closely related to the degree of plant damage under oxidative stress [15,16,17]. Transcriptome sequencing provides a powerful tool to investigate gene expression patterns under waterlogging stress conditions. He et al. demonstrated that the differentially expressed genes (DEGs) in leaf and root tissues of citrus under waterlogging stress were predominantly enriched in distinct pathways. Among them, transcription factors (TFs) AP2/ERF, bHLH, and HSF may be involved in regulating the expression of DEGs in leaves, while HSFs, EREBP, and WRKYs may mainly regulate the expression of DEGs in roots [18]. Transcriptome analysis of *zmrafs*-1 in maize revealed that the genes associated with galactose metabolism and the auxin biosynthetic pathway were up-regulated by raffinose under waterlogging treatment [19]. Dossa et al. conducted high-resolution transcriptome sequencing analysis on the control group and two contrasting sesame genotypes under waterlogging and drainage treatments. They identified 47 waterlogging-responsive genes in sesame. Among them, the transcriptional changes induced by waterlogging/drainage were primarily regulated by ERF and WRKY transcription factors (TFs) [20,21]. By analyzing the transcriptomes of different genotypes, potential key genes regulating resistance to waterlogging in sesame can be identified.

Sesame (*Sesamum indicum* L.), an essential oilseed crop, holds a pivotal position in the agricultural economy of many countries, including China, renowned for its abundant oil and protein content in its seeds [22,23]. Currently, the global cultivation area spans approximately 12.84 million hectares, with an annual production of around 6.74 million tons [24]. However, despite its inherent advantages, the current sesame varieties exhibit numerous issues, including inadequate resistance to disease and stress, low yield, and instability. Sesame growth is highly susceptible to environmental stresses, particularly waterlogging stress, a major threat to its growth and productivity [25,26]. Therefore, a highly promising approach is the development of waterlogging-resistant, high-yield varieties. Waterlogging stress inflicts damage upon the root system of sesame plants while impeding their growth and development processes, ultimately leading to reduced crop yield and quality. In severe cases of waterlogging-induced stress on sesame crops, yields can decrease by over 50%, with complete harvest failure occurring during extreme years [27,28]. Moreover, a majority of sesame genotypes in the field are unlikely to survive beyond 36 h following exposure to waterlogging stress [29]. During the sesame seedling stage, 48 h of waterlogging stress adversely affected sesame growth and significantly impacted its survival after 10 days [30].

Currently, research on sesame waterlogging resistance mainly focuses on the morphological structures and physiological mechanisms. However, investigations at the molecular level remain inadequate. The increased waterlogging and associated crop losses highlight the urgency of understanding plant waterlogging responses and tolerance mechanisms. Determining the molecular mechanism of sesame response to waterlogging stress is crucial for developing effective strategies to enhance its tolerance and improve crop yield, as well as ensuring sustainable production. In this study, two distinct sesame varieties, C177 (waterlogging-tolerant) and C185 (waterlogging-sensitive), were selected for investigation based on the previous results of field waterlogging treatment [31]. Physiological analyses were conducted, and transcriptome sequencing was performed to investigate the gene expression profiles of sesame at the full-bloom stage under waterlogging treatment. The research aims to understand the temporal dynamic changes in gene expression and reveal the molecular mechanism of sesame waterlogging response.

## 2. Results

### 2.1. Effects of Waterlogging Stress on Sesame Physiological and Biochemical Parameters

**Figure 1:**
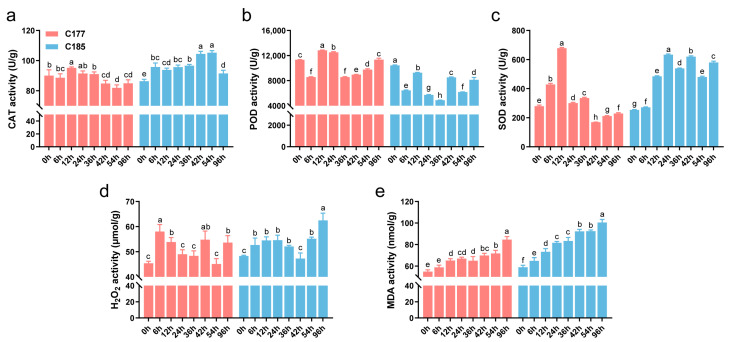
Effects of waterlogging stress on the physiological and biochemical parameters of sesame. The activity of (**a**) CAT, (**b**) POD and (**c**) SOD and the content of (**d**) H_2_O_2_ and (**e**) MDA in waterlogged sesame plants were compared to the untreated plants. Error bars represent the mean ± SD (*n* = 3) of three independent biological replicates. Different lowercase letters indicate statistically significant differences at *p* < 0.05.

Waterlogging stress often induces oxidative damage in plants, and several physiological indices (CAT, POD, SOD, MDA, H_2_O_2_) were investigated in this study (Figure 1). The results demonstrated that waterlogging stress significantly affected the accumulation of MDA and H_2_O_2_. Compared to the untreated control, the levels of MDA and H_2_O_2_ in both genotypes were significantly increased, with C185 showing higher MDA content than C177 after treatment. The activity of antioxidant enzymes showed distinct trends under waterlogging stress. Specifically, the activities of CAT and SOD in C177 initially increased and then decreased, while in C185, the activities of CAT and SOD were significantly higher after treatment, indicating an up-regulation. In contrast, the POD activity in C185 was consistently lower than the control after treatment. These findings suggest that different antioxidant enzymes may exhibit varying responses to waterlogging stress, and there are also some differences among varieties. Additionally, C185 showed greater oxidative damage, indicating a potential difference in waterlogging resistance between C177 and C185.

### 2.2. Identification of Differentially Expressed Genes Under Waterlogging Treatment

**Figure 2:**
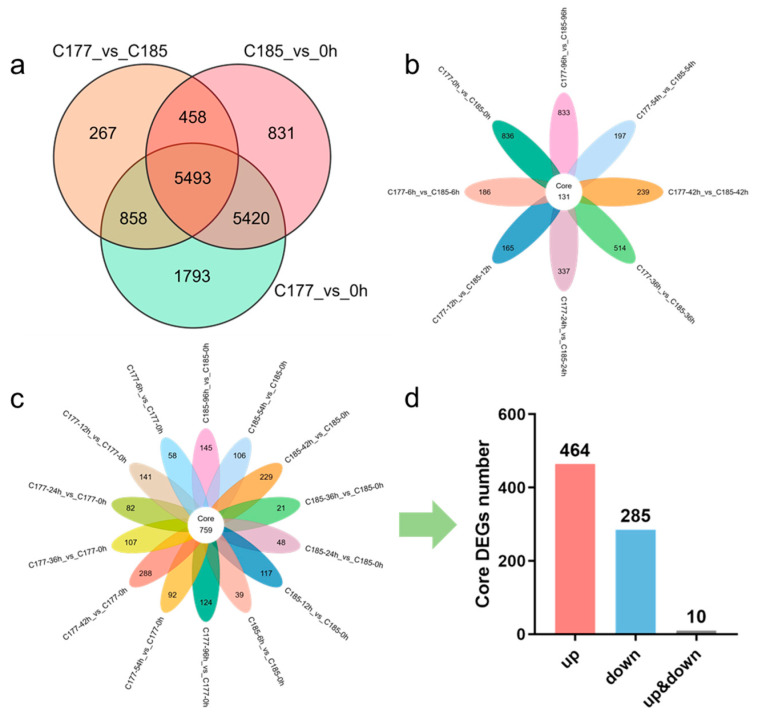
Identification of DEGs in different groups under waterlogging stress. (**a**) Venn diagram analysis of common DEGs in different groups. (**b**) Venn diagram analysis of common DEGs in group C177_vs_C185. (**c**) Venn diagram showing core DEGs in group C177_vs_0 h and C185_vs_0 h. (**d**) The number of core DEGs in group C177_vs_0 h and C185_vs_0 h.

Due to the extreme sensitivity of sesame to waterlogging, and to investigate how waterlogging stress influences gene expression in sesame, we conducted a comprehensive study on two sesame varieties (C177 and C185) during a 96 h period of waterlogging treatment, based on the existing tolerance identification results. Root samples were collected at eight consecutive time points, and transcriptome sequencing was performed with three biological replicates for each sample. A total of 312.27 Gb of clean data were obtained from 48 cDNA libraries after filtering low-quality reads and adapter sequences, with each sample yielding over 5 Gb of clean data. The Q20 and Q30 base percentages were consistently above 97.94% and 94.01%, respectively, indicating high quality of the sequencing data. The alignment efficiency between clean reads and the reference genome exceeded 90.34% (Table S2), producing 25,407 genes, highlighting effective utilization of the transcriptome data. Gene expression levels were normalized using fragments per kilobase of transcript per million fragments mapped (FPKM), and differentially expressed genes (DEGs) were identified based on stringent criteria: |log2Fold change| ≥ 1 and false discovery rate (FDR) < 0.05. A total of 15,143 DEGs were identified (13,564 genes in group C177_vs_0 h, 12,202 genes in group C185_vs_0 h, and 7076 genes in group C177_vs_C185). There were 10,913 common DEGs between the two genotypes, while C177 had 2651 specific DEGs and C185 had 1289 specific DEGs (Figure 2a). Furthermore, principal component analysis (PCA) was conducted, revealing that the samples could be roughly divided into four distinct groups (0 h, 6 h, 12 h, and 24–96 h) (Figure S1).

To identify potential genes related to waterlogging stress, we selected the samples at the 0-h time point as controls (C177_vs_0 h and C185_vs_0 h) and analyzed the DEGs identified in the two genotypes. The results showed that the trends in the number of DEGs in C177 and C185 were highly consistent, with a gradual increase from 6 h to 42 h followed by a gradual decrease, and both up-regulated and down-regulated DEGs exhibiting similar patterns of change (Figure S2a–c). Notably, significant differences in DEG numbers were observed at 36 h in C177_vs_0 h, C185_vs_0 h and C177_vs_C185 (Figure S2a,d). These findings indicate that a large number of genes in sesame were significantly affected by waterlogging stress, with a pronounced difference observed at 36 h and 42 h, which suggests that these time points hold great significance in the response to waterlogging stress. In addition, we identified a total of 759 genes that were differentially expressed across all time points under waterlogging treatment in both genotypes (Figure 2c). Among them, 464 DEGs exhibited up-regulation while 285 DEGs showed down-regulation (Figure 2d, Table S3a). We proposed that these genes may play key roles in the response to waterlogging stress. Additionally, we identified 131 common DEGs (54 up-regulated and 74 down-regulated) that were consistently differentially expressed in C177_vs_C185, which may be associated with the waterlogging tolerance in sesame (Figure 2b, Table S3b).

### 2.3. GO (Gene Ontology) and KEGG (Kyoto Encyclopedia of Genes and Genomes) Enrichment Analysis of Core DEGs in Group C177_vs_0 h and C185_vs_0 h

**Figure 3:**
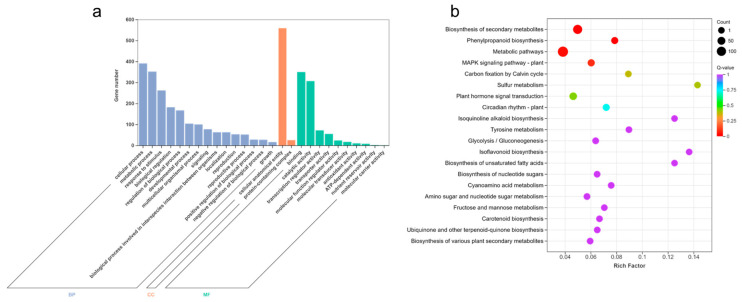
GO enrichment and KEGG pathway analyses of core DEGs under waterlogging stress. (**a**) GO enrichment analysis of core DEGs related to waterlogging stress. (**b**) KEGG pathway analysis of core DEGs related to waterlogging stress.

In order to analyze the functions of 759 core DEGs under waterlogging stress, we conducted GO and KEGG enrichment analyses. The DEGs were divided into three groups: biological process (BP), cellular component (CC), and molecular function (MF) (Figure 3a, Table S4a). Among them, response to decreased oxygen levels (GO:0036293), response to oxygen levels (GO:0070482), cellular response to hypoxia (GO:0071456), response to hypoxia (GO:0001666), and cellular response to decreased oxygen levels (GO:0036294) were highly enriched in BP. Moreover, apoplast (GO:0048046), plant-type vacuole membrane (GO:0009705), and transcription repressor complex (GO:0017053) showed significant enrichments in CC as well. In terms of MF, carboxylic acid binding (GO:0031406), organic acid binding (GO:0043177), heme binding (GO:0020037), oxidoreductase activity, acting on paired donors, with incorporation or reduction of molecular oxygen (GO:0016705), and monocarboxylic acid binding (GO:0033293) were highly enriched.

Additionally, KEGG enrichment analysis demonstrated that a total of 258 DEGs were mapped to the KEGG pathways, with coding genes assigned to 97 KEGG pathways (Table S4b). Notably, biosynthesis of secondary metabolites (ko01110), glycolysis/gluconeogenesis (ko00010), phenylpropanoid biosynthesis (ko00940), metabolic pathways (ko01100), MAPK signaling pathway-plant (ko04016), carbon fixation by Calvin cycle (ko00710), and plant hormone signal transduction (ko04075) exhibited significant enrichment (Figure 3b).

### 2.4. GO (Gene Ontology) and KEGG (Kyoto Encyclopedia of Genes and Genomes) Enrichment Analysis of Common DEGs in Group C177_vs_C185

The results of physiological index determination indicated potential differences in waterlogging tolerance between C177 and C185. We conducted a comparative analysis of the libraries from the two genotypes in the same period and identified 131 common DEGs. These DEGs may be associated with sesame resistance. Moreover, we also performed GO and KEGG analyses on these DEGs (Table S5a,b). The GO enrichment results (Figure S3a) showed that in the biological process category, DEGs related to the regulation of cytoplasmic transport (GO:1903649), regulation of early endosome to late endosome transport (GO:2000641), early endosome to late endosome transport (GO:0045022), vesicle-mediated transport between endosomal compartments (GO:0098927), and amyloplast organization (GO:0009660) terms were significantly enriched. In the cellular component category, DEGs involved in the late endosome membrane (GO:0031902), late endosome (GO:0005770), peroxisome (GO:0005777), and microbody (GO:0042579) were significantly enriched. In the molecular function category, DEGs associated with protein phosphatase regulator activity (GO:0019888), phosphatase regulator activity (GO:0019208), myo-inositol transmembrane transporter activity (GO:0005365), and abscisic acid binding (GO:0010427) were also significantly enriched.

Moreover, the KEGG enrichment analysis (Figure S3b) revealed that the DEGs were significantly enriched in the pathways including nucleotide metabolism (ko01232), plant-pathogen interaction (ko04626), and pyruvate metabolism (ko00620).

### 2.5. Identification of Differentially Expressed Transcription Factors

**Figure 4:**
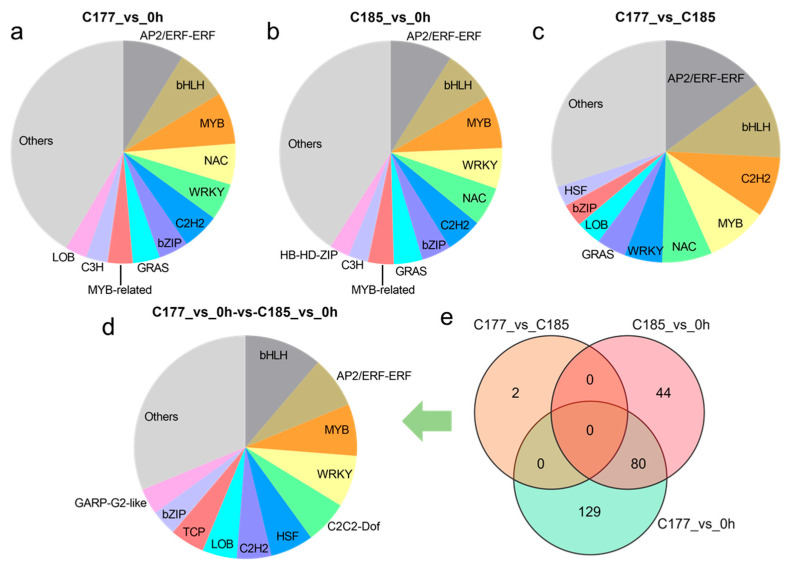
Analyses of the most enriched TF families under waterlogging stress. (**a**) C177_vs_0 h, (**b**) C185_vs_0 h, (**c**) C177_vs_C185, (**d**) C177_vs_0 h-vs-C185_vs_0 h. The bar plot shows the ratios of different TF families. (**e**) Venn diagram analysis of common TFs in different groups.

Transcription factors (TFs), as special proteins regulating gene transcription activities, play a crucial role in plant responses to biotic and abiotic stresses. The transcription factor prediction analysis revealed that the identification of 1026, 935, and 579 TFs in groups C177_vs_0 h, C185_vs_0 h and C177_vs_C185, respectively, which were classified into 62 TF families (Table S6). Among these TFs, the AP2/ERF, bHLH, MYB, NAC, WRKY, and C2H2 families were the main TFs detected throughout the waterlogging stress (Figure 4a–c). Further analysis showed that 209 TFs in C177 and 124 TFs in C185 exhibited continuous differential expression under waterlogging stress. In addition, the core genes responding to waterlogging stress included 80 TFs (Figure 4e), with the largest number of members belonging to the bHLH (9), AP2/ERF (6), MYB (6), and WRKY (6) families (Figure 4d, Table S6d). In the comparison between C177 and C185 at the same time points, only two TFs, *Sin01472* and *Sin27693*, were identified, and both TFs showed consistently down-regulated expression at all time points (Figure 4e, Table S6e).

### 2.6. Weighted Gene Co-Expression Network Analysis

**Figure 5:**
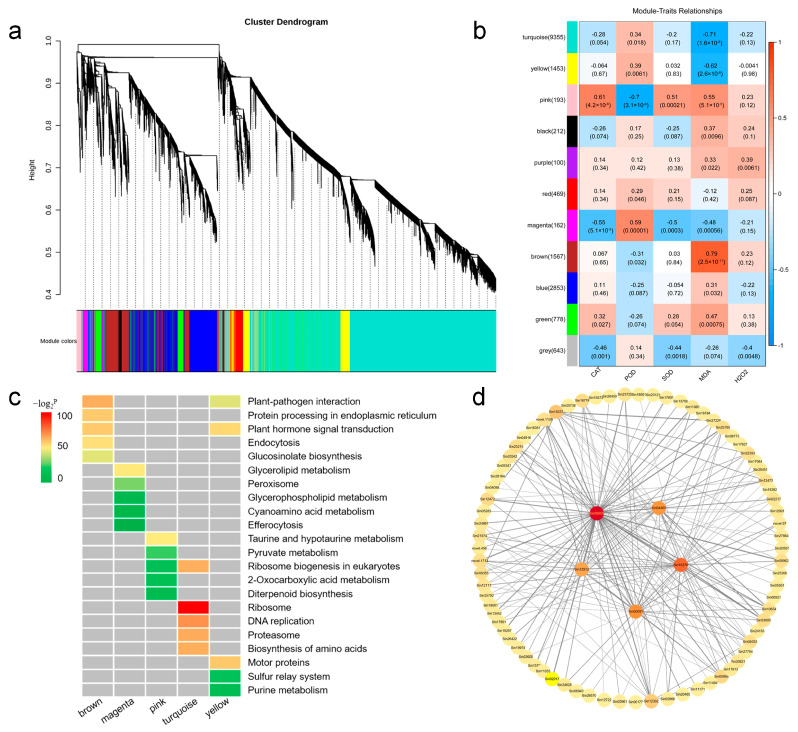
Correlation analysis of waterlogging stress-responsive genes in sesame. (**a**) Dendrogram indicating co-expression modules analyzed by WGCNA under waterlogging stress. (**b**) Heatmap showing correlations between gene modules and physiological indexes. Different colors represent different gene modules. Red color and blue color indicate positive and negative correlation between the gene clusters and the physiological indexes respectively. (**c**) KEGG pathway analysis of the top five enrichment pathways of the genes in different modules. (**d**) Co-expression network analysis in brown module.

To further investigate the connection between genes and waterlogging stress, we conducted weighted gene co-expression network analysis (WGCNA) to construct the co-expression networks based on all genes. Our results showed that 17,785 genes were classified into eleven distinct modules according to their expression patterns, with each module containing 100 to 9355 genes (Figure 5a,b), respectively. Compared with other modules, for example, the brown module exhibited a significant positive correlation with the MDA content (*r* = 0.79). Conversely, the turquoise (*r* = −0.71) and “yellow” modules (*r* = −0.62) showed significant negative correlations with MDA content. Additionally, the pink (*r* > |0.51|) and magenta modules (*r* > |0.48|) displayed meaningful correlations with CAT, POD, SOD activities, and MDA content (Figure 5b). Therefore, this result suggests that these modules may play important roles in the response to waterlogging stress in sesame.

To understand the biological function within different modules, we applied KEGG pathway enrichment analysis to identify enriched pathways in the brown, turquoise, yellow, pink, and magenta modules (Table S7). Among these modules, the brown module was significantly enriched in plant-pathogen interaction, protein processing in endoplasmic reticulum, plant hormone signal transduction, endocytosis, and glucosinolate biosynthesis pathways. The turquoise module exhibited significant correlations with ribosome, DNA replication, proteasome, biosynthesis of amino acids, and ribosome biogenesis in eukaryotes. The yellow module was predominantly associated with motor proteins, plant hormone signal transduction, and plant-pathogen interaction (Figure 5c and Figure S4).

Gene co-expression networks can be employed to reveal the correlations and interactions among genes. The node represents a gene, and the connecting lines (called edges) between nodes signify gene co-expression correlations. Genes with the highest connectivity are termed hub genes, which may play important roles in different modules. We have screened out the brown module and selected the top 200 gene pairs with the highest weights within this module for data visualization. Finally, five genes with the highest connectivity among them are determined as hub genes (Figure 5d). The genes were listed in Table S10.

### 2.7. K-Means Clustering

**Figure 6:**
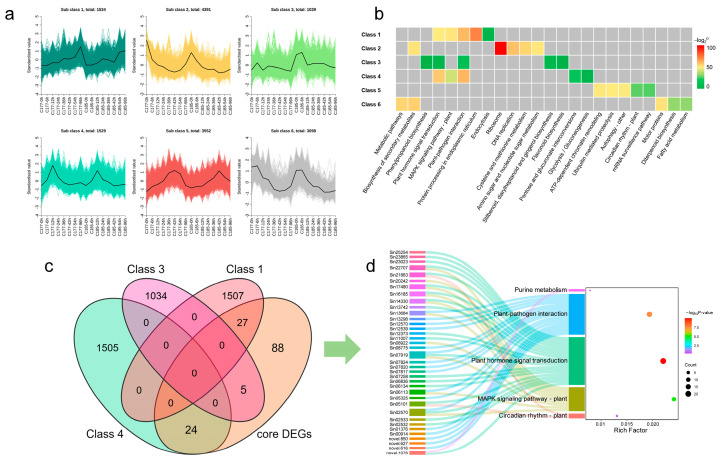
Hierarchical clustering of the DEGs among the sixteen libraries. (**a**) K-means clustering of all DEGs (*k* = 6). (**b**) KEGG pathway analysis of the top five enriched pathways of the DEGs in different clusters. Significance in each cluster is indicated using −log_2_^*p*-value^. The grey areas represent the missing values. (**c**) Venn diagram analysis of common DEGs in different groups. (**d**) Sankey diagram analysis of candidate genes and KEGG enriched pathways. The left rectangle represents gene IDs, the middle rectangle displays the enriched KEGG pathways. Significance is indicated using the −log_10_^*p*-value^.

The K-means clustering was used to analyze the DEGs, with the *k* value set to 6. Finally, a total of 15,143 genes were selected and classified into six distinct classes (Figure 6a). In two genotypes, all six clusters exhibited similar expression patterns. Specifically, class 1 (1534) showed an overall up-regulated expression trend; class 2 (4391) and class 6 (3098) displayed down-regulated expression, whereas class 3 (1039), class 4 (1529), and class 5 (3552) presented an up-regulated then down-regulated trend. To explore the main enriched pathways of DEGs in different clusters, we conducted KEGG pathway analysis and listed the top five most confident pathways in each cluster (Figure 6b, Table S8). The results showed that under waterlogging stress, while the expression patterns of genes in different clusters exhibited similarities, there were significant differences in the enriched pathways. The most significantly affected pathways across the six clusters were as follows: protein processing in endoplasmic reticulum (class 1), ribosome (class 2), plant-pathogen interaction (class 3 and class 4), ATP-dependent chromatin remodeling (class 5), and biosynthesis of secondary metabolites (class 6). In addition, plant hormone signal transduction and MAPK signaling pathway-plant were also significantly enriched in class 1, class 3, and class 4; this result was similar to that derived from the WGCNA analysis. Therefore, we focused on the genes significantly enriched in the plant hormone signal transduction, MAPK signaling pathway-plant, and plant-pathogen interaction pathways, combined with core DEGs and TFs, and finally identified 56 candidate genes (Figure 6c,d, Table S9), which include 25 TFs and 32 DEGs.

### 2.8. Quantitative Real-Time PCR Validation

To validate the reliability of RNA-seq data, we selected ten DEGs and performed quantitative real-time PCR (qRT-PCR) analysis. The results demonstrated that qRT-PCR expression patterns were highly consistent with RNA-seq data, showing a good positive correlation (*R^2^* = 0.9099) (Figure S5), confirming the accuracy of transcriptome data in this study.

## 3. Discussion

As a severe abiotic stress, waterlogging has been constantly impacting the food supply and economic stability of certain regions [32]. Waterlogging leads to a complex series of changes in environmental parameters, causing hypoxia and high carbon dioxide concentrations in the root zone, which influences gas exchange [33]. This is often accompanied by the increased mobilization of toxic substances, which impact plant metabolism, water and nutrient uptake, growth, and even death. Since hypoxia inhibits respiration, plants exhibit both metabolic and morphological adaptations to respond to energy shortages under hypoxic conditions [5,34,35]. Waterlogging tolerance has been an important breeding goal worldwide for over four decades, and breeding waterlogging-resistant varieties has been a top priority for plant breeders [36]. Through crossing tolerant and high-yielding varieties, a rice variety with high yield potential and waterlogging tolerance was successfully developed in 1993 [37]. In the 1970s, Chinese sesame breeders discovered that hybrid vigor could improve the waterlogging tolerance of sesame varieties to some extent, and they initiated research on the morphology, structure, and physiological mechanisms of sesame waterlogging tolerance in the 1990s [38].

Transcriptome sequencing offers an effective strategy for unraveling the gene expression regulatory network in plants under waterlogging stress. Relevant research has been reported across various plant species [39,40,41,42]. Furthermore, multiple genes associated with waterlogging resistance, such as *SUB1A*, *SNORKEL1*, *SNORKEL2*, *ZmEREB180*, *WRKY40*, and *WRKY45*, have been identified [43,44,45,46]. However, the potential molecular mechanisms of sesame under waterlogging stress remain limited. In this study, we investigated the physiological and biochemical characteristics of two sesame genotypes under waterlogging stress. Additionally, transcriptome sequencing and qRT-PCR were employed to analyze gene expression profiles and enrichment pathways. A total of 15,143 DEGs were identified, with 10,913 common DEGs shared between the two genotypes. Overall, 759 genes were continuously differentially expressed under waterlogging stress, including 465 up-regulated DEGs and 285 down-regulated DEGs. These genes are involved in metabolic response to hypoxia, oxidoreductase activity, plant hormone signal transduction and other processes, playing crucial roles in maintaining cellular homeostasis and may represent the core genes that respond to waterlogging stress in sesame. The GO enrichment analysis showed that these DEGs were most enriched in cellular processes, metabolic processes, binding, and catalytic activity. These findings are consistent with the previous study on sesame [47]. Furthermore, the KEGG pathway analysis revealed that the core DEGs were significantly enriched in several key pathways, including metabolic, biosynthesis of secondary metabolites, glycolysis/gluconeogenesis, phenylpropanoid biosynthesis, and plant hormone signal transduction. These pathways exhibit high similarity across different species under the waterlogging stress [18,20,47,48]. Through WGCNA analysis, we identified and further screened five hub genes, which include two F-box genes, two bZIP transcription factors, and an exocyst component protein (Table S10).

Waterlogging stress induces a series of responses and cross-signaling pathways within the plant, including hormones, O_2_, NO, ROS, energy, and other signals, to help plants adapt to waterlogging stress [8,49,50,51,52]. In this study, the activities of antioxidant enzymes and the contents of MDA and H_2_O_2_ in two sesame varieties showed significant changes, especially the content of MDA, which gradually increased with the treatment time. These findings are similar to those of previous studies. The differences in antioxidant enzyme activities indicate that the response of the plant antioxidant system to stress is highly complex and dynamic. These enzymes may have functional differentiation and be regulated by multiple factors [20]. Through module-traits relationships analysis, we identified a module that exhibited a significant positive correlation with MDA and screened two F-box genes (*Sin09950* and *Sin12912*) as hub genes. F-box proteins mediate protein degradation through the S-phase kinase-associated protein 1 (SKP1)-cullin 1 (CUL1)-F-box protein (SCF) complex and are widely involved in both biotic and abiotic stresses [53]. Besides, transcriptome data indicated that a large number of DEGs were enriched in pathways related to these signals. For instance, response to hypoxia, glycolysis/gluconeogenesis, phenylpropanoid biosynthesis, plant-pathogen interaction, and plant hormone signal transduction pathways were significantly affected. IAA (indole-3-acetic acid), ABA (abscisic acid), and ETH (ethylene), as key hormones within the plant, play important roles in plant adaptation to water stresses. ABA cooperates with hormones such as IAA to regulate root growth and development, thereby enabling plants to adapt to osmotic stress. Ethylene can trigger a series of physiological and morphological adaptive changes for plant response to waterlogging stress [54,55,56]. The WGCNA and K-means clustering analysis showed that numerous genes are related to the plant hormone signaling pathways, and we identified several key genes in this pathway, such as probable indole-3-acetic acid-amido synthetase GH3.1 (*Sin02532*/*Sin02533*) and abscisic acid receptor PYL4 (*Sin16185*). Relative research on *Arabidopsis thaliana* has revealed that ABA signals mediated by *MYB96* are integrated into an auxin signaling pathway that encompasses a specific subset of *GH3* genes, which encode auxin-conjugating enzymes to regulate drought stress [56]. Furthermore, overexpression of *AtPYL4* significantly enhances drought tolerance in *A. thaliana* [57]. This points to the potential importance of these genes in relation to waterlogging stress. These findings suggest that sesame employs a complex network of signaling pathways involving multiple hormones and other signals to cope with waterlogging stress, highlighting the importance of understanding these regulatory mechanisms for improving plant tolerance.

As pivotal regulators in plant gene expression, TFs significantly influence plant growth, development, and responses to environmental stresses [58,59,60,61,62]. Accumulating evidence indicates that some TFs play a key role in plant responses to waterlogging stress. For example, the ERF-VIII family member *CmERF4* negatively regulates chrysanthemum waterlogging tolerance by modulating energy metabolism and the ROS pathway genes [63]. In *Arabidopsis*, an allele of *WRKY22* was identified, featuring a two-nucleotide deletion in its promoter region, which confers enhanced adaptability to waterlogged conditions [64]. Previous studies on sesame have revealed that WRKY and ERF TFs are closely related to waterlogging and drainage response. Moreover, WGCNA analyses have identified the potential roles of hub genes in modulating responses to waterlogging stress, including WRKY, NAC, and C2H2 [7,20]. In this study, a total of 62 TF families were found to be differentially expressed in response to waterlogging stress, with the most abundant members belonging to AP2/ERF, bHLH, MYB, NAC, and WRKY families. The AP2/ERF family remains one of the most crucial TFs in regulating response to waterlogging stress, and this result was further verified in our study. Furthermore, our results also highlight the potential significant contributions of bHLH and MYB TFs in the waterlogging response. Members of these families are likely to play crucial regulatory roles under waterlogging stress. Combined with K-means clustering and WGCNA analysis, 27 TFs were identified, like bZIP (*Sin04465*, *Sin00091*), AP2/ERF (*Sin07942*, *Sin26814*), and bHLH (*Sin14330*, *Sin21863*), as well as WRKY (*Sin01376*, *Sin06113*). These TFs were all significantly up-regulated after waterlogging stress. Among them, *WRKY33* has recently been identified as regulating acclimation to submergence in *Arabidopsis* [65].

Based on our findings, we developed a molecular mechanism model delineating the response of sesame to waterlogging (Figure S6). According to this model, waterlogging stress induces differential expression of a series of transcription factors (AP2/ERF, bHLH, bZIP, and WRKY) and genes associated with pathways involved in metabolism, hypoxia/stimulus response, glycolysis/gluconeogenesis, antioxidant enzyme activity, plant hormone signal transduction, and plant-pathogen interaction. These alterations may lead to physiological and biochemical modulations, such as antioxidant enzyme activity and the contents of H_2_O_2_ and MDA. Ultimately, these modifications may confer adaptability to waterlogging stress in sesame.

## 4. Materials and Methods

### 4.1. Plant Materials and Growth Conditions

The two sesame varieties, Yanzhou Erhong Pi (C177, waterlogging-tolerant) and Yuzhi No.8 (C185, waterlogging-sensitive), were cultivated in the controlled greenhouse at the Henan Academy of Agricultural Sciences, China. Prior to sowing, the sesame seeds underwent disinfection treatment as follows: immersed in 75% alcohol for 1 min, rinsed with sterile water, treated with 10% NaClO for 5 min, and then washed 5–6 times with sterile water. Twelve seeds were planted in each PVC pot, with a ratio of nutritious soil to loam soil of approximately 2:1. After germination, only four healthy and uniform plants per pot were retained through thinning. The growth conditions were set to a day/night temperature of 30/25 °C, a photoperiod of 16 h light/8 h dark, a light intensity of 350 µmol·m^−2^·s^−1^, and a relative humidity of 70%.

### 4.2. Plant Treatment and Sampling

When seedlings grow into the full-bloom stage, the experimental group was subjected to waterlogging by placing them in plastic buckets filled with tap water, with the water level maintained at about 3 cm above the soil surface, and kept waterlogged for 96 h. Meanwhile, the control group remained under normal growth conditions throughout the entire experiment. The roots (whole root) of C177 and C185 were collected in three biological replicates at eight time points: 0 h, 6 h, 12 h, 24 h, 36 h, 42 h, 54 h, and 96 h after waterlogging treatment. The samples were immediately frozen in liquid nitrogen and stored at −80 °C until further use.

### 4.3. Physiological Index Measurement

In this study, the catalase (CAT) activity was determined using the CAT Activity Assay Kit (ammonium molybdate-chromogenic method); the peroxidase (POD) activity was measured by the POD Activity Assay Kit (guaiacol method); the superoxide dismutase (SOD) activity was assessed with the SOD Activity Assay Kit (nitrogen blue tetrazolium photo reduction method); the malondialdehyde (MDA) content was quantified through the MDA Content Assay Kit (thiobarbituric acid method); and the content of hydrogen peroxide (H_2_O_2_) was detected by the H_2_O_2_ Content Assay Kit (titanous sulfate method). All detection kits were purchased from Solarbio Science and Technology (Beijing, China) and used according to the manufacturer’s protocol.

### 4.4. Transcriptome Analysis

RNA-seq library construction and subsequent analysis were conducted at Metware Technologies (Wuhan, China) using the Illumina platform. The clean data were obtained by removing adapters and reads with low quality and then mapped to the reference genome of Yuzhi No. 4. Subsequently, String Tie (https://ccb.jhu.edu/software/stringtie/ (accessed on 8 August 2024)) was utilized for estimating transcript levels and calculating fragments per kilobase of transcript per million fragments mapped (FPKM) values [66,67]. DESeq2 was used for differentially expressed genes (DEGs) analysis [68,69], with the Benjamini-Hochberg method applied to adjust *p*-values for multiple hypothesis testing in order to control the false discovery rate (FDR). Adjusted |log_2_ (fold change)| ≥ 1 and *p*-value < 0.05 were set as thresholds for screening significant DEGs. Functional annotation of DEGs was conducted through Gene Ontology (GO) enrichment analysis and Kyoto Encyclopedia of Genes and Genomes (KEGG) enrichment [70,71]. Additionally, principal component analysis (PCA), weight gene co-expression network construct and analysis (WGCNA), and K-means cluster analysis were performed on the Metware Cloud platform (https://cloud.metware.cn/ (accessed on 13 September 2024)). The DEG co-expression networks were visualized using Cytoscape 3.10.3.

### 4.5. RNA Extraction and qRT-PCR Validation

RNA extraction and quantitative real-time PCR (qRT-PCR) validation were performed following the methods described below. The total RNA of root samples was isolated using RNAiso Plus (Takara, Beijing, China, Code No. 9108) according to the manufacturer’s protocol. RNA quality was assessed through agarose gel electrophoresis and NanoPhotometer^®^ spectrophotometry (IMPLEN, Westlake Village, CA, USA). High-quality RNA was then reverse-transcribed into cDNA using the MightyScript Plus First Strand cDNA Synthesis Master Mix (with gDNA digester) (Sangon Biotech, Shanghai, China, Order No. B639252). To validate the sequencing results, ten DEGs were selected for qRT-PCR analysis. The qRT-PCR reactions were performed using the SYBR Green system (Vazyme, Nanjing, China, Code No. Q712), with the *SiHistone* (*Sin27220*) gene serving as the endogenous control [72]. The relative expression levels were calculated using the 2^−ΔΔCT^ method. All specific primers utilized in the qRT-PCR assay were designed based on the National Center for Biotechnology Information (NCBI) database (www.ncbi.nlm.nih.gov/tools/primer-blast (accessed on 20 October 2024)), and their sequences are listed in Table S1.

### 4.6. Statistical Analysis

All treatments were replicated three times both technically and biologically, and all data were subjected to statistical analysis. Graphs were generated using GraphPad Prism 8.0.2 software. Statistical analysis was conducted using IBM SPSS 26.0 software with Duncan’s test (*p* < 0.05), and differences at *p* < 0.05 and *p* < 0.01 were considered significant and highly significant, respectively.

## 5. Conclusions

In summary, the transcriptomic profiles and physiological parameters of two sesame genotypes under waterlogging stress were analyzed in this study. It was found that waterlogging stress induced oxidative damage and affected the activity of antioxidant enzymes. A total of 759 core DEGs were identified, which were differentially expressed continuously at all time points. The enriched pathways of DEGs were analyzed via GO and KEGG analyses. Moreover, the transcription factor families such as AP2/ERF, bHLH, bZIP, and WRKY might play pivotal roles in the sesame response to waterlogging stress. Combined with K-means clustering and WGCNA analysis, 56 genes and 5 hub genes were identified as potential candidate genes in the response to waterlogging stress. These findings not only enhance our understanding of the genetic basis underlying sesame’s molecular response to waterlogging stress but also provide valuable molecular markers and candidate genes for sesame breeding programs aimed at improving waterlogging tolerance.

## Data Availability

Data will be made available on request.

## Supplementary Materials

The following supporting information can be downloaded at: https://www.mdpi.com/article/10.3390/ijms26062603/s1.
