# Supplementary material for: Physiological and Transcriptional Responses of Sesame (Sesamum indicum L.) to Waterlogging Stress
Source: Int J Mol Sci. 2025 Mar 13;26(6):2603. doi: 10.3390/ijms26062603 (PMC11942034; doi:10.3390/ijms26062603)
Supplement: Supplementary file 1 [file ijms-26-02603-s001.zip › Supplementary Figure.pdf]

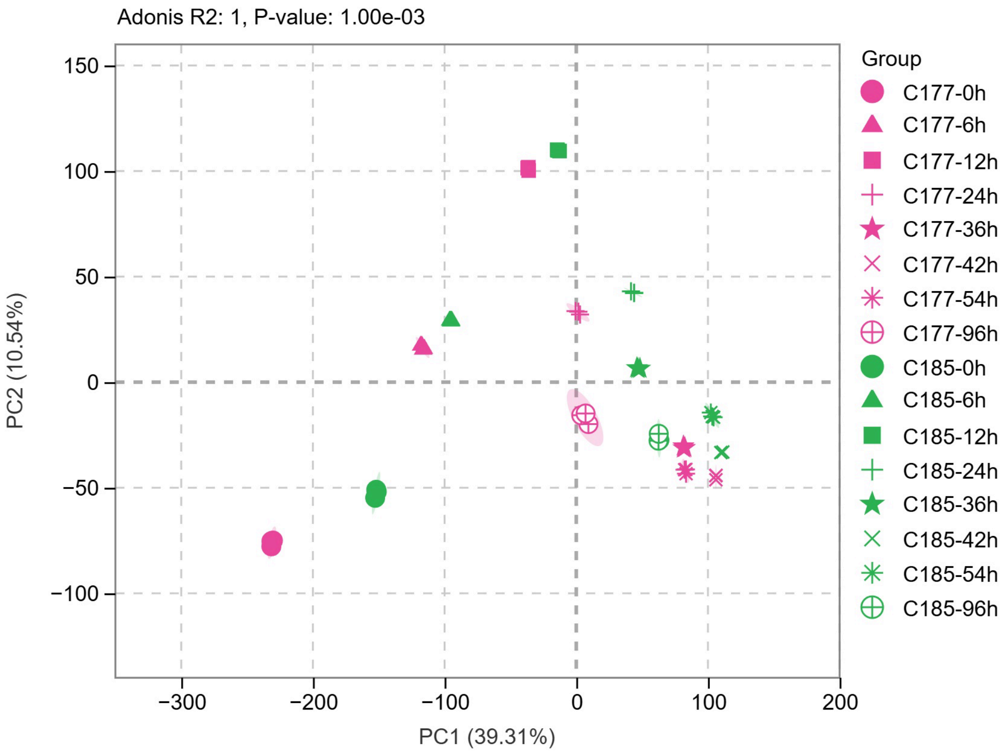

Figure S1. PCA analysis of the genes in C177 and C185.

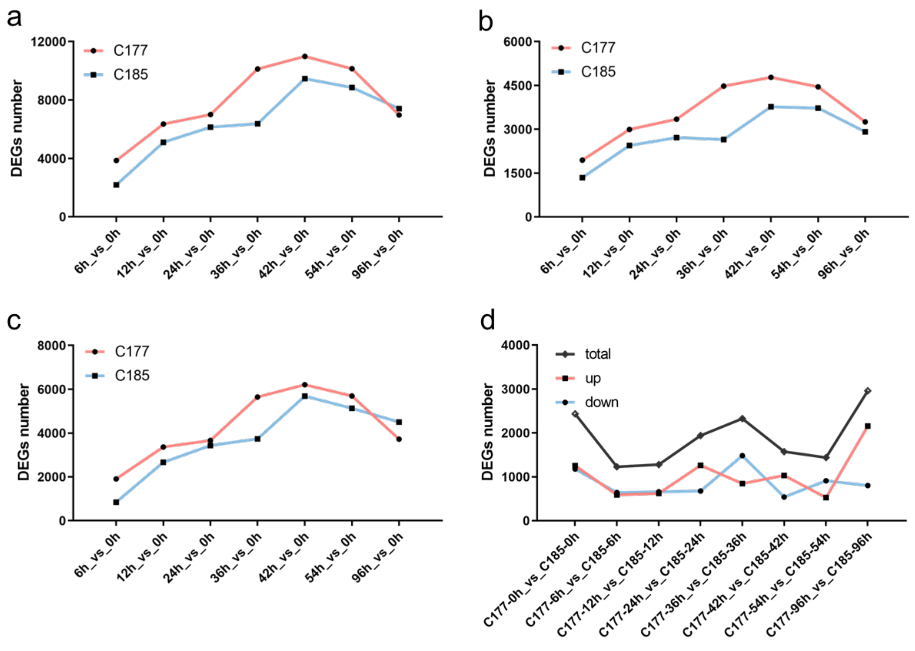

Figure S2. Analyses of DEGs under waterlogging stress. (**a**) Number of all DEGs. (**b**) Number of upregulated DEGs. (**c**) Number of downregulated DEGs in groups C177_vs_0h and C185_vs_0h. (**d**) Number of DEGs in group C177_vs_C185.

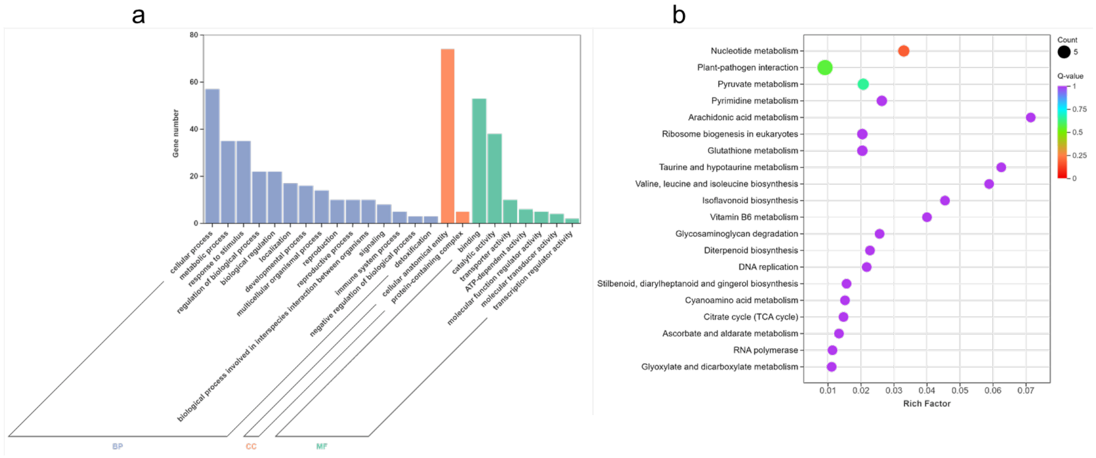

Figure S3. GO enrichment and KEGG pathway analyses of common DEGs under waterlogging stress. (**a**) GO enrichment analysis of common DEGs related to waterlogging resistance. (**b**) KEGG pathway analysis of common DEGs related to waterlogging resistance.

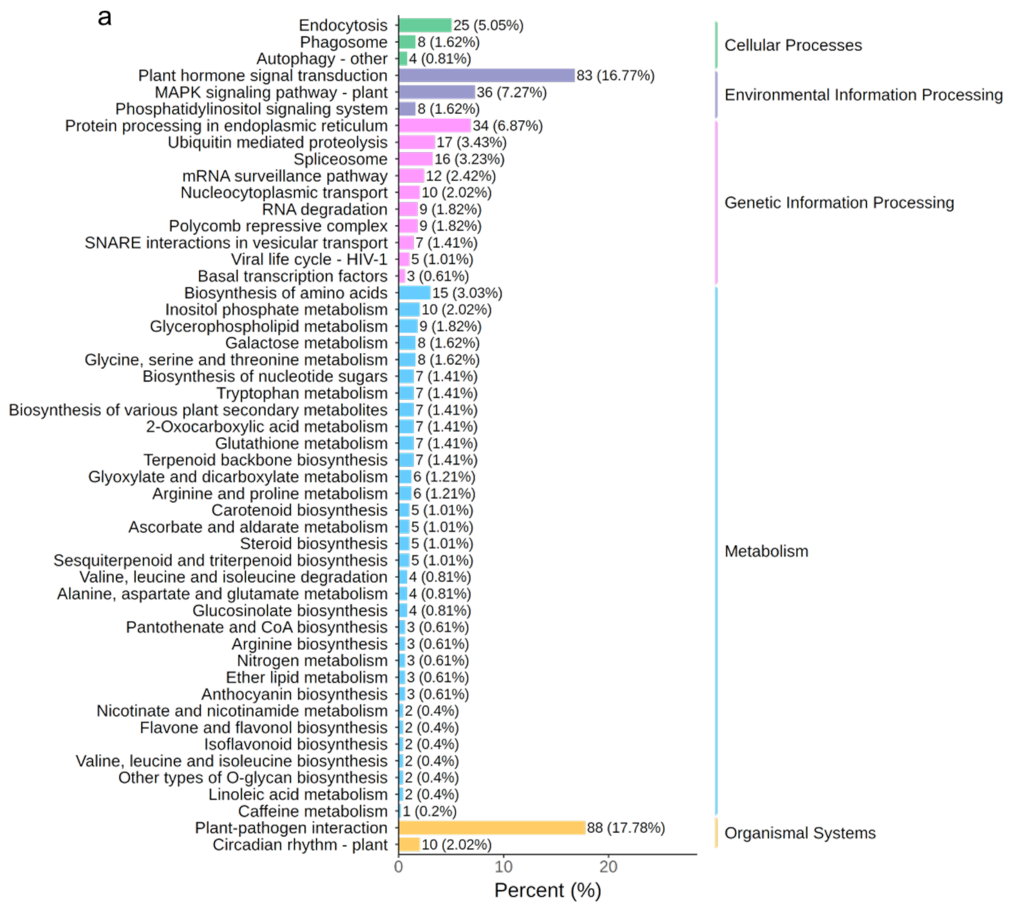

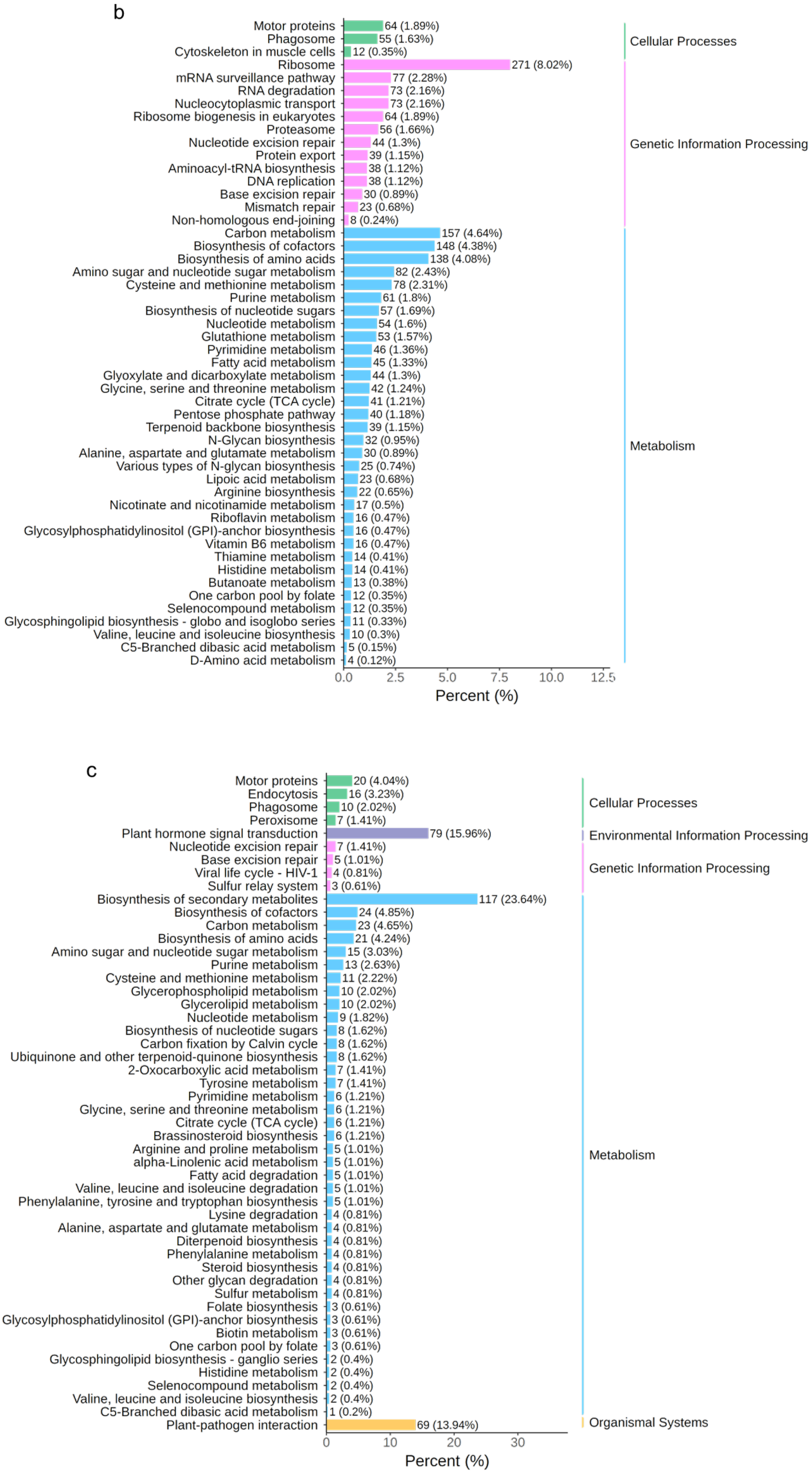

Figure S4. KEGG pathway analyses of (**a**) brown (**b**) turquoise and **(c)** yellow modules.

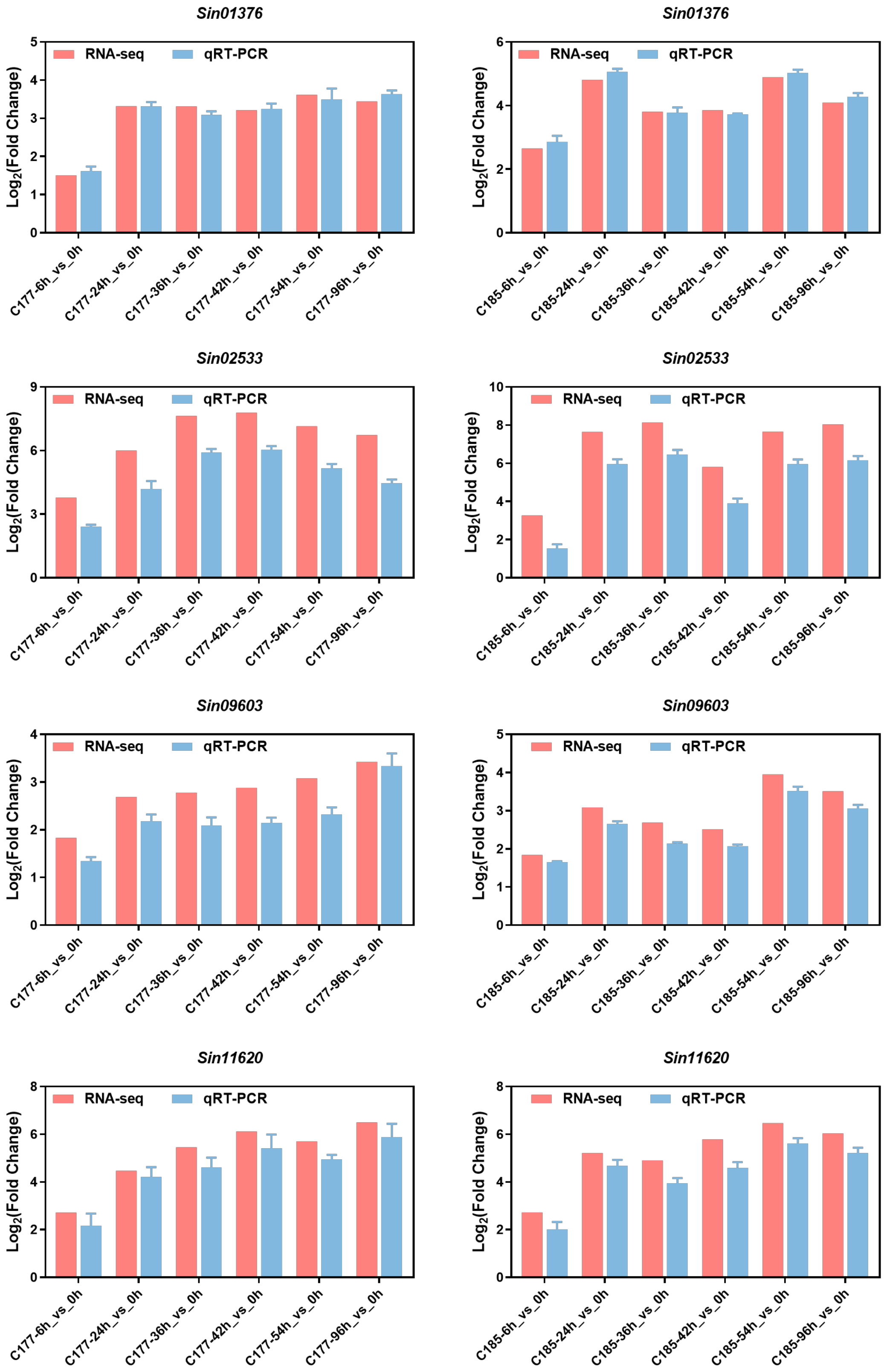
Sin01376
Sin01376
Sin02533
Sin02533
Sin09603
Sin09603
Sin11620
Sin11620
RNA-seq
qRT-PCR
Log2(Fold Change)
C177-6h_vs_0h
C177-24h_vs_0h
C177-36h_vs_0h
C177-42h_vs_0h
C177-54h_vs_0h
C177-96h_vs_0h
C185-6h_vs_0h
C185-24h_vs_0h
C185-36h_vs_0h
C185-42h_vs_0h
C185-54h_vs_0h
C185-96h_vs_0h

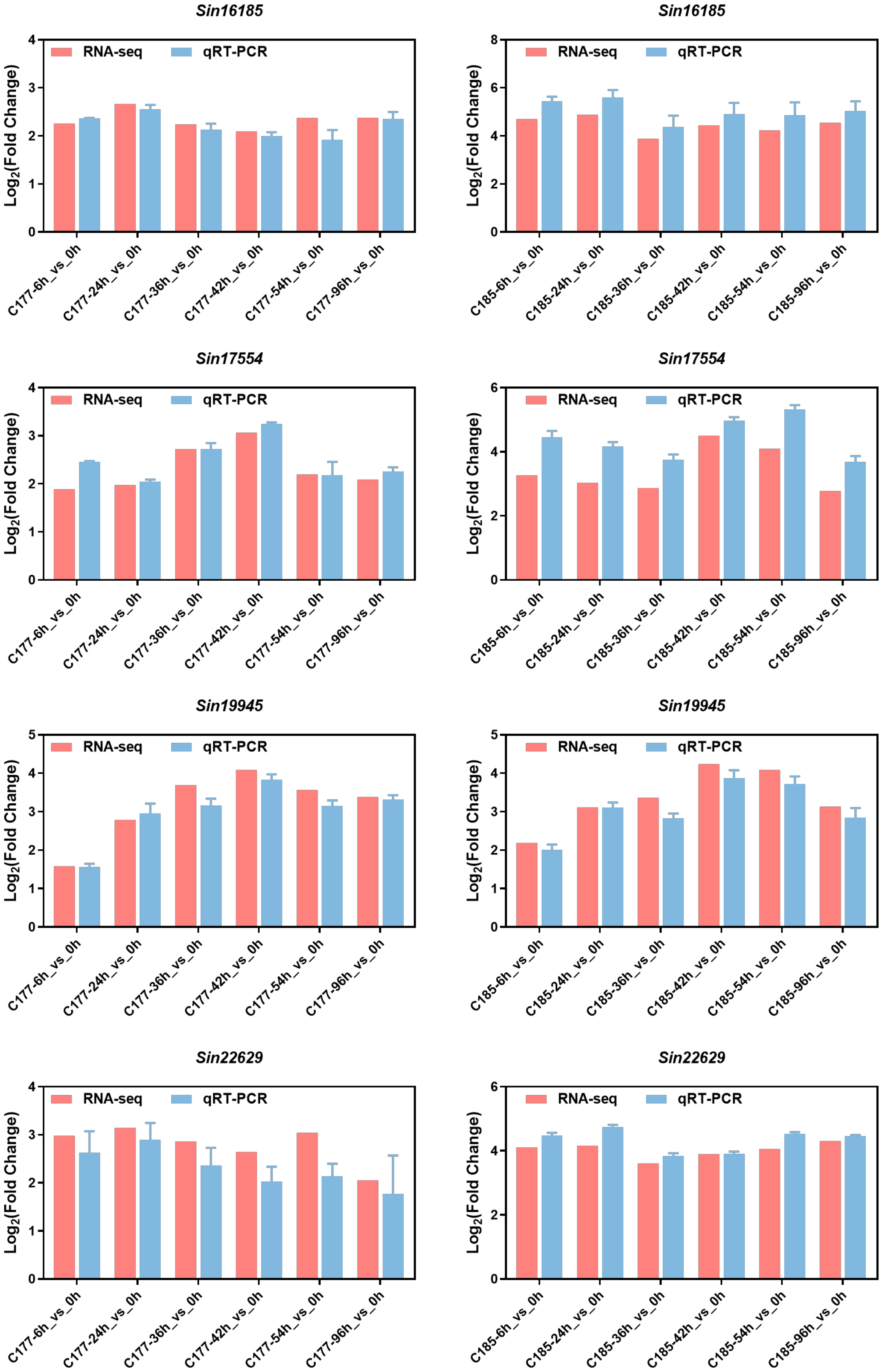
Sin16185
RNA-seq
qRT-PCR
Log2(Fold Change)
C177-6h_vs_0h
C177-24h_vs_0h
C177-36h_vs_0h
C177-42h_vs_0h
C177-54h_vs_0h
C177-96h_vs_0h
Sin16185
RNA-seq
qRT-PCR
Log2(Fold Change)
C185-6h_vs_0h
C185-24h_vs_0h
C185-36h_vs_0h
C185-42h_vs_0h
C185-54h_vs_0h
C185-96h_vs_0h
Sin17554
RNA-seq
qRT-PCR
Log2(Fold Change)
C177-6h_vs_0h
C177-24h_vs_0h
C177-36h_vs_0h
C177-42h_vs_0h
C177-54h_vs_0h
C177-96h_vs_0h
Sin17554
RNA-seq
qRT-PCR
Log2(Fold Change)
C185-6h_vs_0h
C185-24h_vs_0h
C185-36h_vs_0h
C185-42h_vs_0h
C185-54h_vs_0h
C185-96h_vs_0h
Sin19945
RNA-seq
qRT-PCR
Log2(Fold Change)
C177-6h_vs_0h
C177-24h_vs_0h
C177-36h_vs_0h
C177-42h_vs_0h
C177-54h_vs_0h
C177-96h_vs_0h
Sin19945
RNA-seq
qRT-PCR
Log2(Fold Change)
C185-6h_vs_0h
C185-24h_vs_0h
C185-36h_vs_0h
C185-42h_vs_0h
C185-54h_vs_0h
C185-96h_vs_0h
Sin22629
RNA-seq
qRT-PCR
Log2(Fold Change)
C177-6h_vs_0h
C177-24h_vs_0h
C177-36h_vs_0h
C177-42h_vs_0h
C177-54h_vs_0h
C177-96h_vs_0h
Sin22629
RNA-seq
qRT-PCR
Log2(Fold Change)
C185-6h_vs_0h
C185-24h_vs_0h
C185-36h_vs_0h
C185-42h_vs_0h
C185-54h_vs_0h
C185-96h_vs_0h

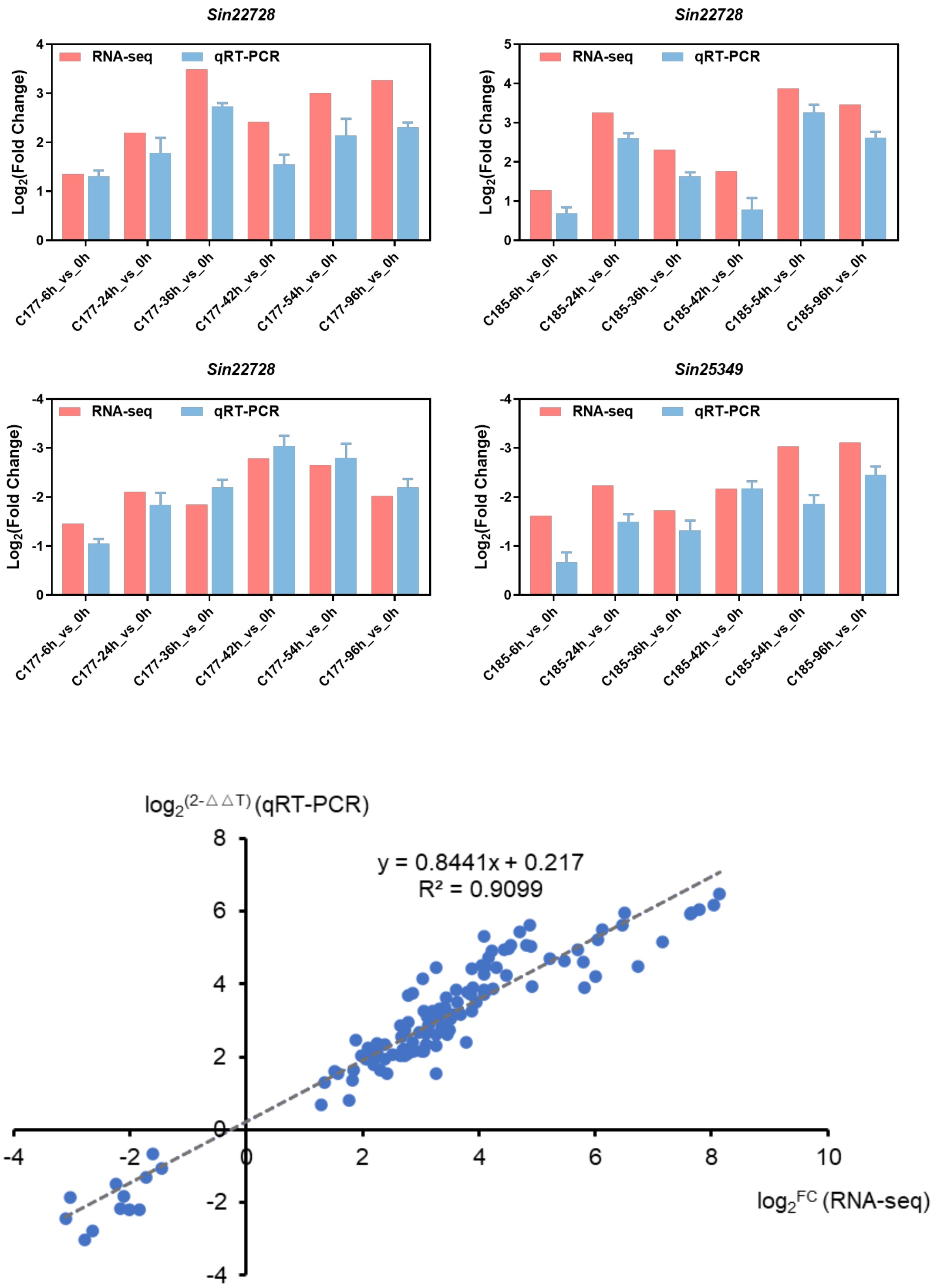

Figure S5. Verification of 10 selected DEGs detected in the RNA-seq. The average data and standard errors were calculated from biological replicates. A Pearson correlation between RNA-seq and qRT-PCR expression profiling was plotted, and data are expressed as a $\log_2$ fold change.

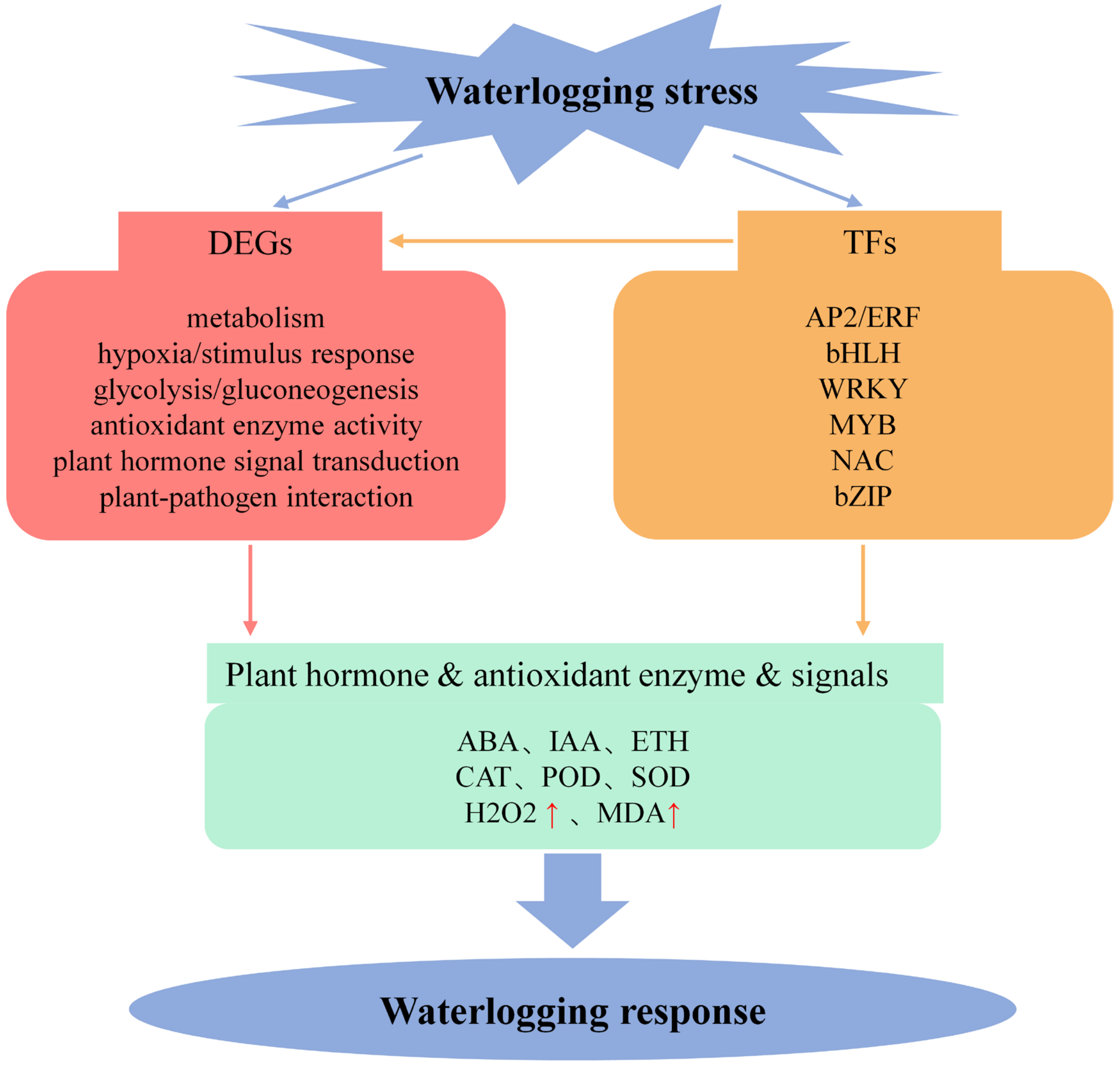

Figure S6. Regulatory model of waterlogging response in sesame.
